# Investigation and Study on the Biology and Morphology of *Apis florea* and *Apis dorsata* in Southern China

**DOI:** 10.3390/life15030341

**Published:** 2025-02-21

**Authors:** Xinying Qu, Xinru Zhang, Tian Sun, Zequn Qiu, Qihuang Lu, Zhenghui Bi, Hanrong Qin, Junjun Hu, Peng Tang, Lianfei Cao, Xiao Chen

**Affiliations:** 1 State Key Laboratory of Resource Insects, Institute of Apicultural Research, Chinese Academy of Agricultural Sciences, Beijing 100193, China; 82101235489@caas.cn (X.Q.); 202330112016@bua.edu.cn (X.Z.); 2 College of Bioscience and Resource Environment, Beijing University of Agriculture, Beijing 102206, China; 3 Beekeeping Guidance Station, Guangxi Zhuang Autonomous Region, Nanning 530012, China; 13558108335@163.com (T.S.); luqih189@163.com (Q.L.); gxyfz5829915@163.com (Z.B.); chsqhr@163.com (H.Q.); 13707719983@163.com (J.H.); tpaengbombus@163.com (P.T.); 4 Beekeeping Guidance Station, Ningming County, Chongzuo 530025, China; 13517670338@163.com; 5 Institute of Animal Husbandry and Veterinary Science, Zhejiang Academy of Agricultural Sciences, Hangzhou 310009, China; caolf@mail.zaas.ac.cn

**Keywords:** honeybees, morphometric, diversity, conservation

## Abstract

Honey bees are crucial pollinators that play a vital role in maintaining ecological balance. The colonies of managed honey bees in China increased rapidly in the past 20 years. Whether the rapid increase in managed bee populations would affect the survival of wild honey bees deserves attention. There are four species of the genus *Apis* in Guangxi, China, including *Apis florea*, *Apis dorsata*, *Apis mellifera ligustica*, and *Apis cerana*. This study conducted an investigation on the biological characteristics of the four species and measured 40 morphological characteristics. The results showed that for the four species, their swarming periods, mating periods, the emergence of drones, and the nectar and pollen source plants highly overlap. This leads to potential competition for the optimum mating space, nectar, and pollen. The comparison of morphological traits between samples collected in 2012 and 2023 showed that the aspect ratios of the forewings of both *Apis florea* and *Apis dorsata* have significantly increased. This change may be attributed to the rapid increase in managed honey bees’ populations, which has encroached upon the living spaces of *A. florea* and *A. dorsata*. The results of coefficient of variations showed that *Apis florea* and *Apis dorsata* exhibit high genetic diversity. Our results indicated that the increased colonies’ number of managed honey bees has an effect on *Apis florea* and *Apis dorsata*, but they are not facing great threats to their survival. Continuous monitoring of the the diversity of *Apis florea* and *Apis dorsata* must be maintained. Preserving wild honey bees is of great significance for the ecological balance.

## 1. Introduction

Honey bees are important pollinators in nature and are indispensable for the ecological balance [1,2,3]. Pollination by honey bees improves the production and quality of crops [4,5,6]. Also, bee products, including honey, royal jelly, and pollen, are good for human health [7,8,9,10]. It is precisely because of the importance of honey bees that the colonies of commercial honey bees in China have increased rapidly over the past 20 years [11]. However, whether the rapid increase in commercial honey bees populations in China would affect the survival of wild honey bees deserves attention.

Previous studies reported that commercial honey bee (*Apis mellifera*) colonies may compete with native bees for flower resources [12,13,14,15,16]. Although commercial honeybees are generally not known to exhibit aggression towards other bees, numerous reports suggest that they deplete the pollen and nectar resources of native plants, leading to a negative correlation between the presence of commercial honeybees and native bees, particularly in areas with high concentrations of commercial bee colonies [16,17]. MacInnis et al. (2023) found substantial evidence for a decrease in wild bee richness following a massive (>12×) increase in honey bee colony density over <10 years [18]. Since honey bees may outcompete and threaten native flower-visiting insects, some countries have made rules to restrict the number and density of commercial honey bee colonies [19,20]. There is strong evidence showing that honey bees compete with native insects in European cities and in areas with high densities of commercial honey bee colonies [21,22].

Located in southern China, Guangxi borders the Beibu Gulf in the south and is crossed by the Tropic of Cancer in its central region. Guangxi boasts a warm climate, abundant sunshine, plentiful rainfall, and rich nectariferous plants, which provide an ideal habitat for honey bees. Four species of the genus *Apis* can be found in Guangxi, including *Apis florea* (*A. florea*), *Apis dorsata* (*A. dorsata*), *Apis mellifera ligustica* (*A. m. ligustica*), and *Apis cerana* (*A. cerana*). Among them, *A. florea* and *A. dorsata* are wild honey bees, while *A. m. ligustica* and *A. cerana* are managed honey bees. In the past 20 years, the beekeeping industry in Guangxi has undergone a rapid development, marked by a steady increase in breeding populations of *A. m. ligustica* and *A. cerana*. In 1999, the number of colonies kept in Guangxi was 277,200, including 33,300 colonies of *A. m. ligustica* and 243,900 colonies of *A. ceana* [23]. In 2014, the number of colonies was 732,000, including 172,500 colonies of *A. m. ligustica* and 559,500 colonies of *A. cerana* [23]. In 2020, the number of colonies was 921,200, including 231,200 colonies of *A. m. ligustica* and 690,000 colonies of *A. cerana* [23]. Over the past 20 years, the number of colonies of *A. m. ligustica* and *A. cerana* kept in Guangxi has increased by 232%. According to surveys on apiculture in Guangxi, both *A. m. ligustica* and *A. cerana* are distributed throughout the whole region, overlapping with the distribution areas of *A. florea* and *A. dorsata*. However, whether the rapid increase in managed honey bee populations will affect the survival of wild *A. florea* and *A. dorsata* is still unclear.

Therefore, this study conducted an investigation on the biological characteristics of *A. florea*, *A. dorsata*, *A. m. ligustica*, and *A. cerana* in Guangxi to find the similarities of biological characteristics for the four species. Morphological characteristics were measured for the four species to find the differences among them. Further, we compared the morphological characteristics of *A. florea* and *A. dorsata* between 2012 and 2023 to analyze whether their morphological features have changed over the past ten years. Based on a comprehensive analysis of these indicators, the study assessed whether the survival of *A. florea* and *A. dorsata* has been impacted by the rapid increase in populations of managed *A. m. ligustica* and *A. cerana* over the past 20 years.

## 2. Materials and Methods

### 2.1. Survey and Data Acquisition

**Figure 1:**
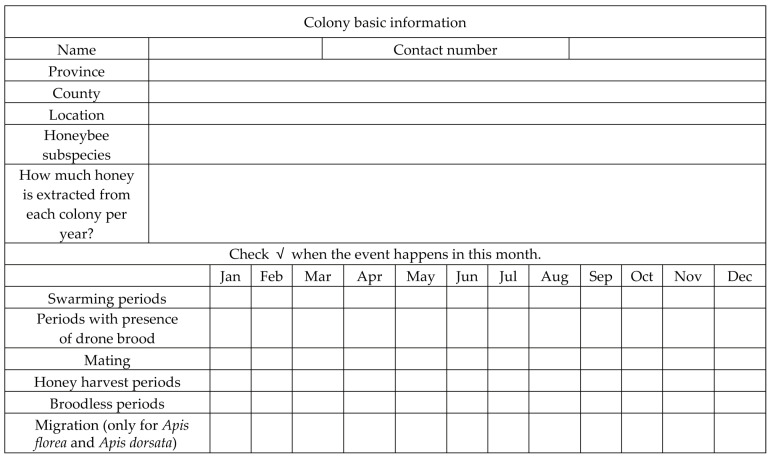
Survey questionnaire for four species of honey bee in the Guangxi region.

We designed a questionnaire to obtain basic biological information about *A. florea*, *A. dorsata*, and *A. m. ligustica* in Guangxi (Figure 1). The biological information of *A. cerana* was from our previous study [24]. Paper copies of the questionnaire were distributed to local beekeeping organizations, which delegated surveyors to randomly select and communicate with beekeepers face-to-face in daily visits. All the completed questionnaires were then returned to our center for data reduction and analysis. Samples of *A. florea* and *A. dorsata* were collected from regions with the highest distribution densities for morphological measurements.

### 2.2. Description of the Area of Study

Guangxi is located in the subtropical monsoon climate zone, with the northern half having a central subtropical climate and the southern half having a southern subtropical climate. Guangxi has a mountainous and hilly basin topography.

In 2021, the average temperature in Guangxi was 21.6 °C, ranging from 17.1 °C to 24.4 °C. In the regions where *A. dorsata* and *A. florea* have been discovered, the extreme highest and lowest temperature over the years is 41.2 °C and −5.2 °C, respectively. In 2021, the average annual precipitation in Guangxi was 1335.3 mm, ranging from 750.7 mm to 2184.5 mm. In 2021, the average annual sunshine hours in Guangxi were 1675.1 h, ranging from 1155.5 h to 2319.6 h. The frost-free period is 327 to 353.7 d.

The distribution area of *A. dorsata* is located between 21°35′ N–24°59′ N and 104°29′ E–110°47′ E in Guangxi. The distribution area of *A. florea* is located between 21°35′ N–25°33′ N, and 104°29′ E–110°53′ E. *A. cerana* and *A. m. ligustica* are kept using modern beekeeping methods in Guangxi.

**Figure 2:**
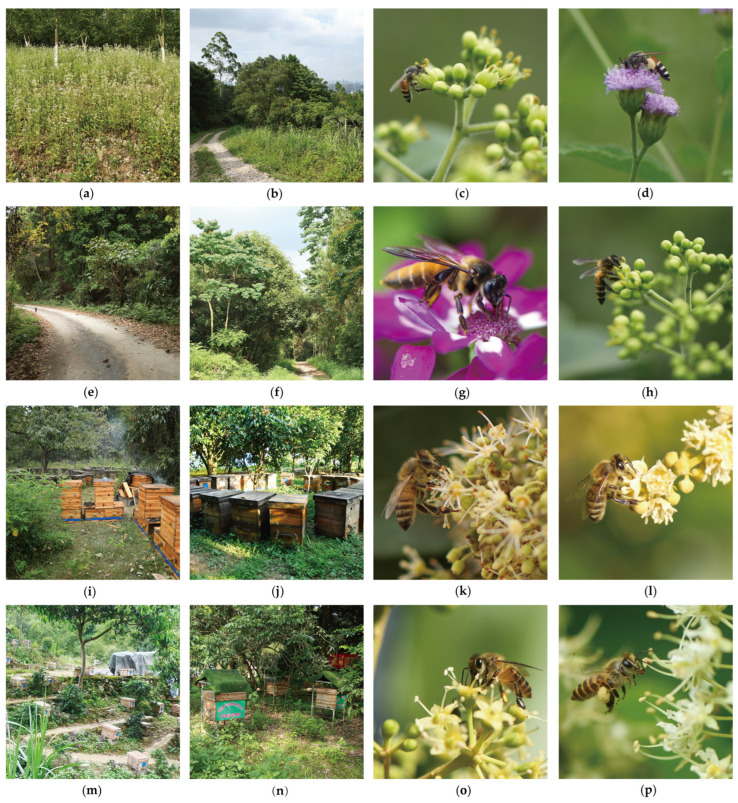
The ecological environments and nectar plants in Guangxi. (**a**,**b**) The ecological environment where the *A. florea* lives. (**c**) *Tetradium ruticarpum* (A. Juss.) T. G. Hartley and *A. florea.* (**d**) *Cyanthillium patulum* (Aiton) H. Rob. and *A. florea.* (**e**,**f**) The ecological environment where the *A. dorsata* lives. (**g**) *Pericallis hybrida* B. Nord. and *A. dorsata*. (**h**) *Tetradium ruticarpum* (A. Juss.) T. G. Hartley and *A. dorsata*. (**i**,**j**) The ecological environment where the *A. m. ligustica* lives. (**k**) *Litchi chinensis* Sonn. and *A. m. ligustica*. (**l**) *Dimocarpus longan* Lour. and *A. m. ligustica.* (**m**,**n**) The ecological environment where the *A. cerana* lives. (**o**) *Heptapleurum heptaphyllum* (L.) Y. F. Deng and *A. cerana*. (**p**) *Phanera championii* Benth. and *A. cerana*.

Previous surveys and research found that there were 735 species of plants in Guangxi pollinated by honey bees [23]. These plants include *Tetradium ruticarpum* (A. Juss.) T. G. Hartley, *Dimocarpus longan* Lour., *Litchi chinensis* Sonn., *Heptapleurum heptaphyllum* (L.) Y. F. Deng, and *Toxicodendron vernicifluum* (Stokes) F. A. Barkley. These nectar and pollen plants bloom continuously throughout the year, providing sufficient nectar and pollen for the survival and development of honey bees (Figure 2) [23].

### 2.3. Sampling and Morphometric Measurements

**Figure 3:**
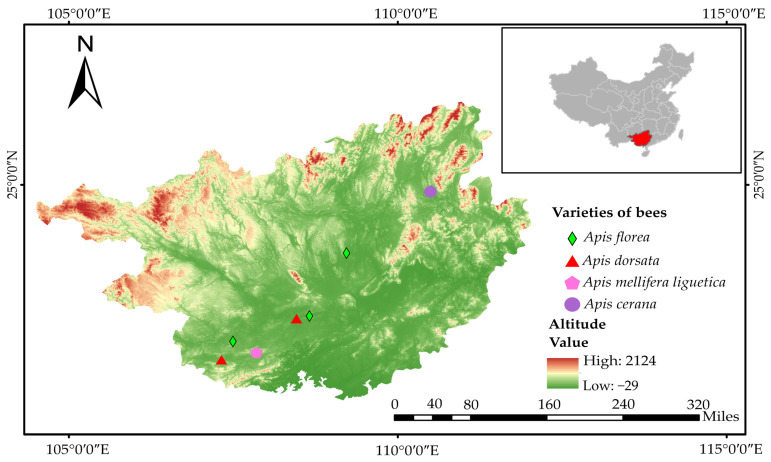
The surveyed area of *A. florea*, *A. dorsata*, *A. m. ligustica*, and *A. cerana*.

In 2023, samples of *A. florea* workers were collected from 10 colonies in 3 locations (Wuhehuaqiao Forest Farm (3 colonies), Jiangzhou Town (5 colonies), and Liangtang Town (2 colonies)) (Figure 3). Samples of *A. dorsata* workers were collected from 10 colonies in 2 locations (Wuxiangling Forest Park (6 colonies) and Dongpo Village (6 colonies)) (Figure 3). Samples of *A. cerana* and *A. m. ligustica* workers were collected from 20 colonies from 2 breeding apiaries (10 colonies for each species). We randomly selected a basket of bees from the hives, preferably from the brood area (which we visually observed to have a strong, fluffy appearance) and a uniform color on the comb. For each colony, around 50 worker bees were collected. We then placed them into a sealed bag and closed it until the bees calmed down or were exposed to the sun for a while. When most of the bees were dead, we transferred them into a 50 mL centrifuge tube and preserved them in 75% ethanol. The collected samples and the morphometric data derived from them are stored at the Institute of Apicultural Research, Chinese Academy of Agricultural Sciences, Beijing, China.

To know the changes of morphometric characteristics of *A. dorsata* and *A. florea* from 2010s to 2020s in Guangxi, morphometric characteristics were measured for samples collected in 2023 and re-measured for samples collected by Lianfei Cao in the same location in 2012 [25]. Morphometric measurements of 40 characteristics were taken from 10 honey bees from each colony, according to the method described by Ruttner and Meixner [26,27]. Measurements of hair were taken under a ZEICS dissecting microscope (Stemi 508) fitted with an eyepiece graticle at a magnification of 32×. Measurements of wings, legs, and sternites were taken with a LEICA DMS 300, which is a Leica microscope with a CCD camera connected to a desktop computer.

### 2.4. Statistical Analyses

The mean, standard deviation, and coefficient of variation in each of the 40 morphometric characteristics were calculated for every colony. The data were subjected to a one-way analysis of variance (ANOVA) to compare results from different species and from different years. Tukey’s HSD tests were used to detect significant differences between the species on the means of the 40 characteristics. The Bonferroni method was used for multiple comparisons to correct the *p*-value. Prior to analysis, the data were assessed for linearity, normal distribution, and the presence of outliers. To analyze the diversity of the four bee populations, the coefficient of variation (CV) was calculated for each bee population using Excel 2007. CV = (standard deviation/average value) × 100%.

## 3. Results

### 3.1. Biological Characteristics

**Figure 4:**
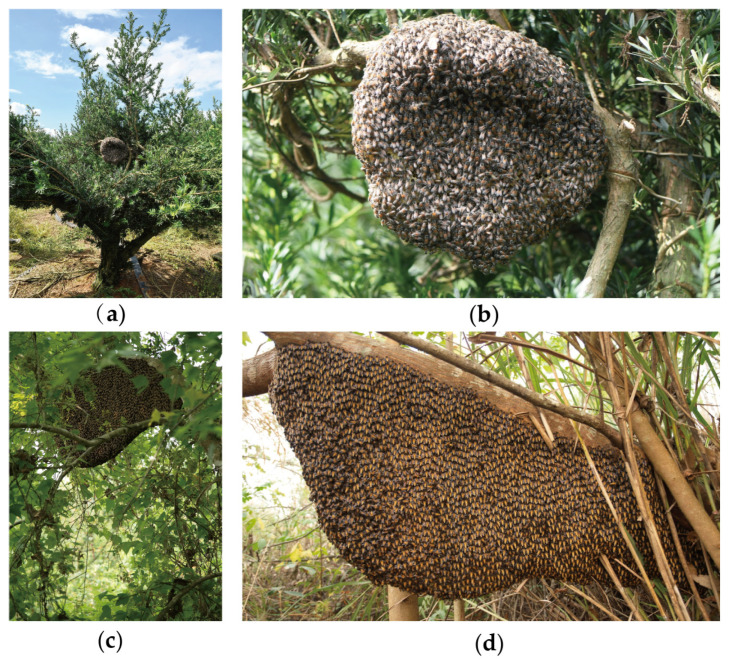
Pictures of *A. florea* and *A. dorsata* colonies. (**a**,**b**) Colony of *A. florea*. (**c**,**d**) Colony of *A. dorsata*.

**Figure 5:**
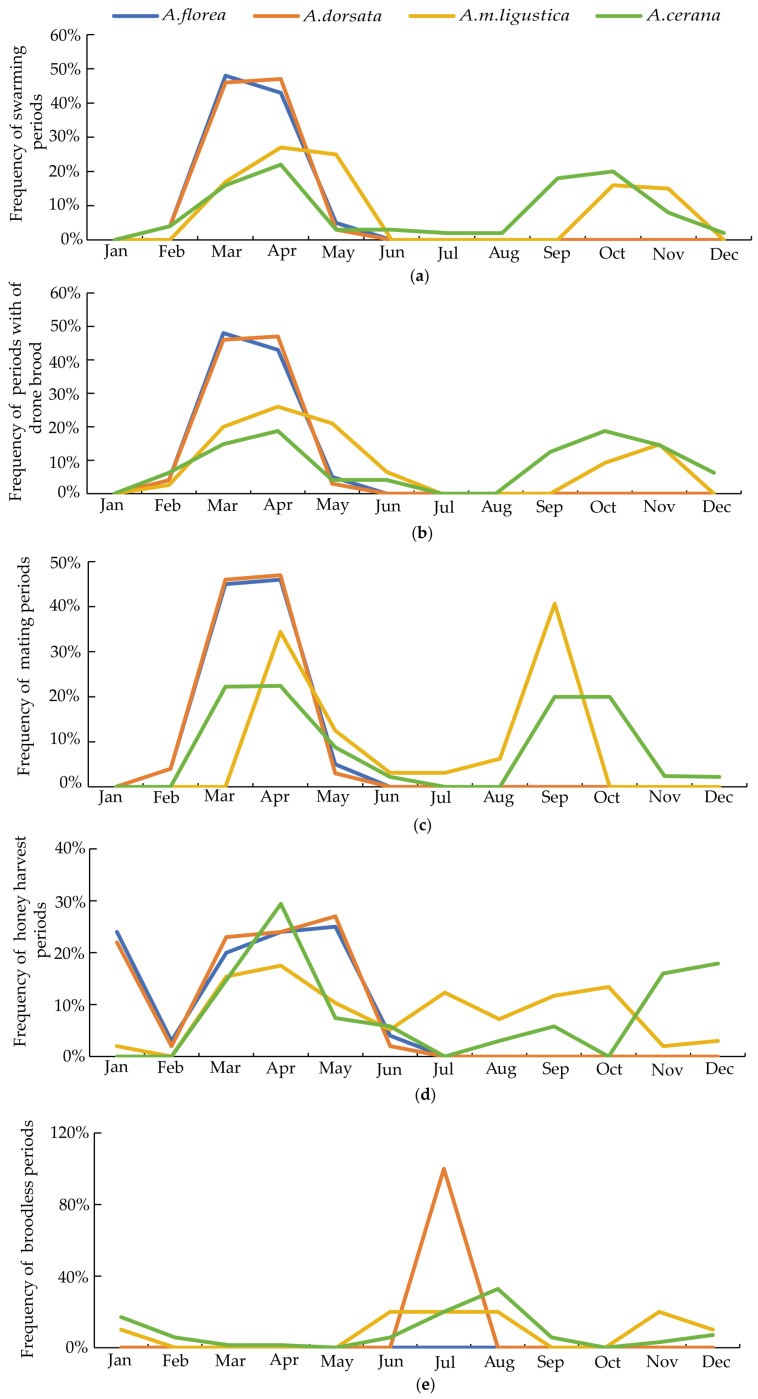
The biological differences among the four types of honey bees: *A. florea*, *A. dorsata*, *A. m. ligustica*, and *A. cerana* in Guangxi region. (**a**) Swarming periods. (**b**) Periods with of drone brood. (**c**) Mating periods. (**d**) Honey harvest periods. (**e**) Broodless periods.

In total, 108 counties (regions) in Guangxi participated in the survey and 412 respondents provided feedback. According to the survey, the migration period of *A. florea* in Guangxi occurs in February and August each year (Figure 4). The emergence of drones, mating time, and swarming period all coincide and are concentrated in March and April (Figure 5). The queen in *A. florea* colonies lays eggs continuously throughout the year without any cessation. In Guangxi, the main honey-harvesting period for *A. florea* is from January to May, with each colony yielding 1–1.5 kg of honey per harvest. No diseases have been observed to date.

The migration of *A. dorsata* in Guangxi occurs annually from February to October (Figure 4). The emergence of drones, mating time, and swarming period all coincide and are mainly concentrated in March and April, similar to that of *A. florea* (Figure 5). Due to the hot weather, the queen of *A. dorsata* stops laying eggs in July and resumes egg-laying in August as the temperature drops. The main honey-harvesting period for *A. dorsata* is from January to May, with each colony yielding 10–25 kg of honey per harvest. No diseases have been observed to date.

In Guangxi, the emergence of drones and swarming in *A. m. ligustica* colonies mainly occur from March to May and from October to November (Figure 5). The queen stops laying eggs from June to August and from November to January of the following year. The main honey-harvesting period is concentrated between March and October each year. In Guangxi, the annual honey production per colony for migratory beekeeping is approximately 60 kg. No occurrences of Sacbrood virus or European foulbrood have been observed.

In Guangxi, *A. cerana* has two main swarming periods, one from March to April and the other from September to November. During these times, drone larvae are formed (Figure 5). This ensures the availability of sufficient mature drones for mating with virgin queens. The queen mainly stops laying eggs in January and from July to August. The main honey-harvesting periods for colonies throughout the year are from May to July, August to September, and November to December, with an annual honey production of approximately 10 to 25 kg per colony.

According to survey results, the nectar and pollen source plants collected by the four species of honey bees in Guangxi are almost the same, mainly including *Litchi chinensis* Sonn., *Dimocarpus longan* Lour., *Triadica cochinchinensis* Loureiro, and *Heptapleurum heptaphyllum* (L.) Y. F. Deng. Competition for the nectar and pollen exists among these four species of honey bee [12,13,14,15,16].

### 3.2. Overall Description of the Morphology of A. florea and A. dorsata

**Figure 6:**
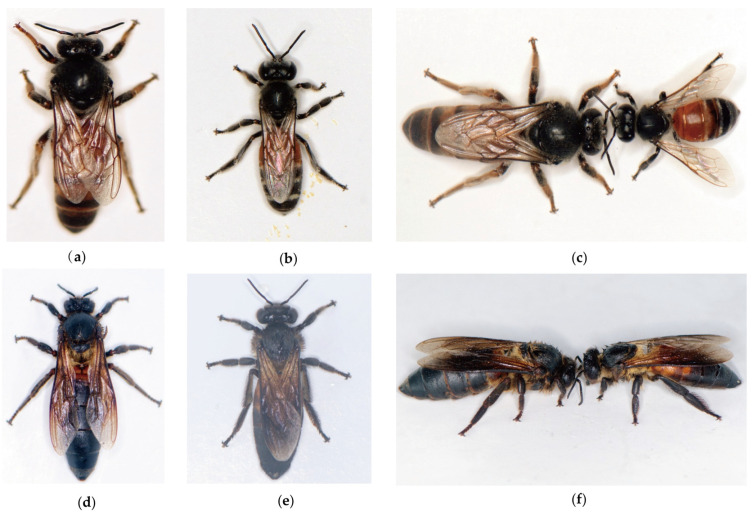
Pictures of *A. florea* and *A. dorsata*. (**a**) The queen of *A. florea*. (**b**) The worker of *A. florea*. (**c**) Top-down view of the queen and worker of *A. florea*, with the queen on the left and the worker on the right. (**d**) The queen of *A. dorsata*. (**e**) The worker of *A. dorsata*. (**f**) Top-down view of the queen and worker of *A. dorsata*, with the queen on the left and the worker on the right.

The queen of *A. florea* measures 16.5 mm in length (Figure 6), with a forewing length of 9.75 mm. The tergites of the first and second abdominal segments, the basal half of the third tergite, and the apical margins of the third to fifth tergites are all reddish-brown, while the rest are black. The worker of *A. florea* measures 7 to 8 mm in length, with the tergites of the first and second abdominal segments being reddish-brown. The head is slightly wider than the thorax, and the apex of the mandibles is reddish-brown. The distance between the two posterior ocelli is greater than the distance from the posterior ocelli to the compound eyes. The length of the malar space is significantly smaller than its width. The scutellum and the tergites of the third to sixth abdominal segments are all black. The body hairs are short and sparse; the face and the lower surface of the head are covered with grayish-white hairs, while the hairs on the vertex are dark brown. The thorax is adorned with grayish-yellow short hairs, and the abdominal tergites are covered with dark brown short hairs. There are white fluffy bands at the bases of the third to fifth tergites. The ventral surface of the abdomen is adorned with fine and long grayish-white hairs. The dorsal sides of both the hind tibia and basitarsus are covered with white hairs on both sides. The drone measures 11 to 13 mm in length and is black in color. It differs from the worker in that the medial lobelike protrusion on the hind tibia is long, slightly exceeding two-thirds of the total length of the tibia.

The queen of *A. dorsata* has a body length of 23 mm (Figure 6), forewing length of 14.5 mm, and is entirely black in color. The drone measures 16 to 17 mm in length. Its compound eyes are large, with the posterior inner edges of the two eyes closely connected. The abdomen is cylindrical and black, with the metapleuron, most of the tergites from the first to the sixth abdominal segments, and the middle and hind legs being reddish-brown. The forelegs are dark brown, and the ratio of the length of the hind tibia to the length of the basitarsus is 5:3. The body hair ranges from light yellow to yellow, with the compound eyes densely covered with short yellow hairs and the clypeus covered with black hairs. The area around the ocelli, cheeks, outer sides of the foreleg coxae, chest, and the first and second abdominal tergites are covered with long yellow hairs. The upper and lower lips of the workers of *A. dorsata* are chestnut-brown, while the first segment of the antennae and the mouthparts are tawny. The hairs on the head are sparse and short, grayish-white in color. The vertex, mesoscutum, and mesopleuron are covered with dense and long dark brown to black hairs. The scutellum and propodeum are adorned with honey-yellow long hairs. The first to third tergites of the abdomen are covered with honey-yellow short hairs, while the remaining segments are adorned with brown to dark brown short hairs. The legs are covered with black hairs. The outer sides of each segment of the forelegs have long yellow hairs, and the inner sides of the basitarsi of the middle and hind legs are adorned with golden-brown hairs. The wings are dark brown with a purple gloss, with the anterior and subanterior cells being the darkest in color, and the hind wings being slightly lighter in shade.

### 3.3. Morphological Changes in A. florea and A. dorsata over the Past Decade

**Figure 7:**
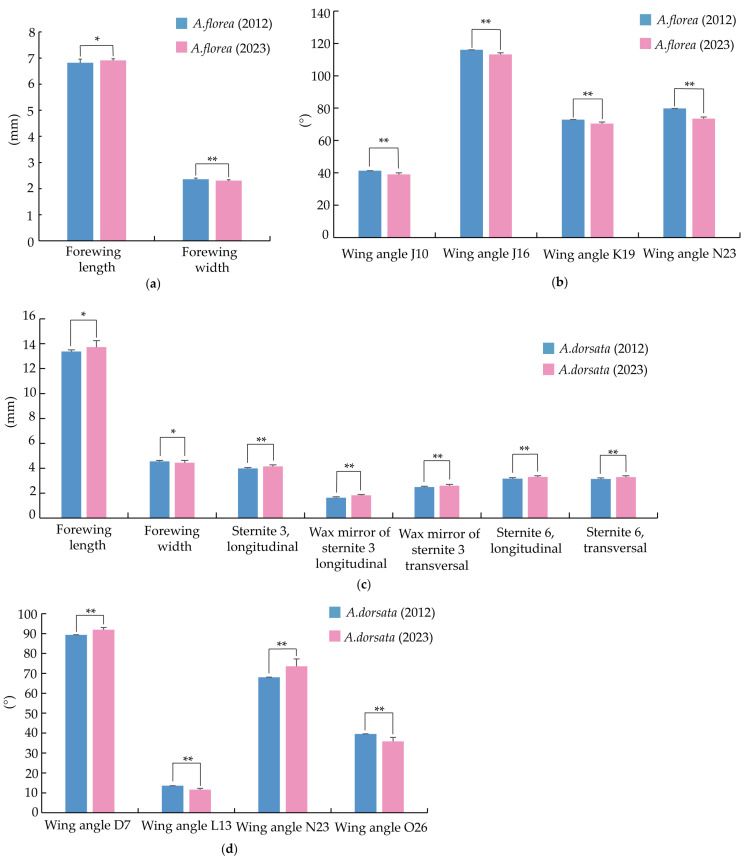
The morphological characteristics that have undergone significant changes in *A. florea* and *A. dorsata* over the past 10 years. (**a**) The forewing length and width of *A. florea* in 2012 and 2023. (**b**) The changed wing angles of *A. florea* in 2012 and 2023. (**c**) The changed morphological characteristics of body size of *A. dorsata* in 2012 and 2023. (**d**) The changed wing angles of *A. dorsata* in 2012 and 2023. Note: *A. florea* (2012) represents the morphological data of the *A. florea* collected in the year 2012. *A. florea* (2023) represents the morphological data of the *A. florea* collected in the year 2023. *A. dorsata* (2012) represents the morphological data of the *A. dorsata* collected in the year 2012. *A. dorsata* (2023) represents the morphological data of the *A. dorsata* collected in the year 2023. * *p* < 0.05.** *p* < 0.01.

To understand the changes of morphometric characteristics of *A. dorsata* and *A. florea* from 2010s to 2020s, morphometric characteristics were compared between samples collected in 2012 and 2023 (Figure 7, Tables S1 and S2). The results showed that over the past decade, the forewing length of *A. florea* has significantly increased by 1.34%, while the forewing width has significantly decreased by 2.30%, resulting in a significant increase in the forewing length-to-width ratio by 3.80%. The wing angles J10, J16, K19, and N23 have significantly decreased by 5.57%, 2.48%, 3.44%, and 7.85%, respectively. For *A. dorsata*, the forewing length has significantly increased by 2.61%, while the forewing width has significantly decreased by 2.28%, leading to a significant increase in the forewing length-to-width ratio of 4.88%. The wing angles D7 and N23 have significantly increased, while L13 and O26 have significantly decreased. The length of the third sternite, the wax mirror of sternite 3 longitudinal, and the wax mirror of sternite 3 transversal have significantly increased by 3.90%, 12.51%, and 4.54%, respectively. The length of the sixth sternite and the width of the sixth sternite have also significantly increased by 4.34% and 4.82%, respectively. These results indicate that significant morphological changes have occurred in *A. florea* and *A. dorsata* over the past decade.

### 3.4. Morphological Comparison of Four Species of Honey Bees in Guangxi

When ranked by body size, the four species of honey bee follow the order *A. dorsata* > *A. m. ligustica* > *A. cerana* > *A. florea*. However, some morphological characteristics do not follow this size variation pattern (Table S1). For example, the hair length of *A. florea* and *A. dorsata* are almost the same. *A. m. ligustica* has the longest hair, measuring 0.23 mm, while *A. cerana* has the shortest hair. The width of stripe posterior of tomentum of *A. dorsata* is the smallest (0.12 mm). The width of stripe posterior of tomentum of *A. cerana* is 0.78 mm, which is the largest among the four honey bee species.

## 4. Variation Analysis of Morphometric Characteristics

**Figure 8:**
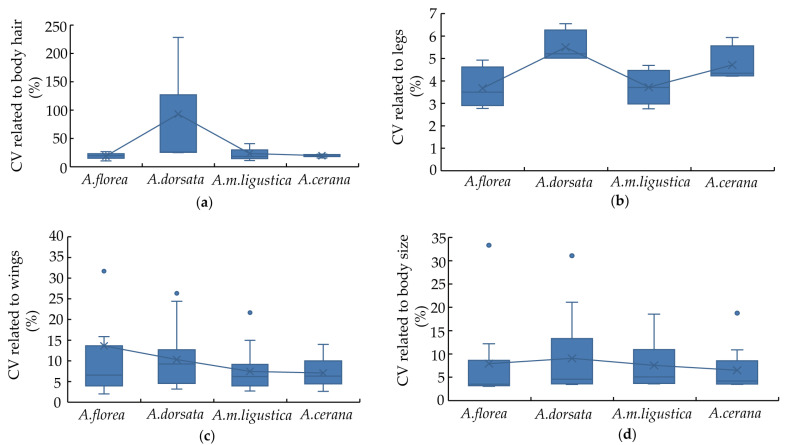
The results of CV for morphological characteristics of *A. florea*, *A. dorsata*, *A. m. ligustic*, and *A. cerana*. (**a**) The CV related to body hair. (**b**) The CV related to legs. (**c**) The CV related to wings. (**d**) The CV related to body size.

By calculating and analyzing the coefficient of variation (CV), it was found that among the characteristics related to hair length (Figure 8a), the average CV for *A. florea* was 18.92%, while for *A. dorsata* it was 93.02%. The average CV for *A. m. ligustica* was 23.40%, and for *A. cerana* it was 19.58%. For the characteristics related to the legs (Figure 8b), the average CV for *A. florea* was 3.68%, while for *A. dorsata* it was 5.5%. For *A. m. ligustica*, it was 3.72% and for *A. cerana* it was 4.71%. Regarding the characteristics related to wings (Figure 8c), the average CV for *A. florea* was 13.60%, for *A. dorsata* it was 10.31%, for *A. m. ligustica* it was 7.43%, and for *A. cerana* it was 7.07%. For the characteristics related to body size (Figure 8d), the average CV for *A. florea* was 7.91%, for *A. dorsata* it was 9.07%, for *A. m. ligustica* it was 7.54%, and for *A. cerana* it was 6.52%.

Among the characteristics related to hair length, the CV for all four species are relatively large, with *A. dorsata* exhibiting the highest average CV, indicating a high degree of variation. For the characteristics related to wings, both *A. florea* and *A. dorsata* have average CVs exceeding 10%, suggesting a significant level of variation. In contrast, the CVs for the characteristics related to legs and body size are relatively small for all four species, indicating stable genetic inheritance. Therefore, *A. florea* and *A. dorsata* possess higher genetic diversity compared to *A. m. ligustica* and *A. cerana*.

## 5. Discussion

Honey bees are important pollinators and play a crucial role in maintaining ecological balance [28,29,30]. Honey bees in China are widely distributed across various geographical and climatic regions [31,32,33]. Through the process of long-term natural selection, each bee species has developed strong adaptability to local ecological conditions. Guangxi, with various honey bee resources, is located in southern China. Nectar and pollen plants are abundant. Plants bloom continuously throughout the year. This provides a natural environment conducive to honey bee survival. Over the past 20 years, the number of colonies of *A. m. ligustica* and *A. cerana* in Guangxi has increased by 230%. Previous studies reported that honey bees compete with native insects, which was particularly strong in European cities and in areas with high densities of commercial hives [18,21,22]. MacInnis et al. (2023) found that honey bee abundance had the strongest negative effect on small (inter-tegular span < 2.25 mm) wild bee species richness [18]. Small bee species may be at higher risk in areas with abundant honey bee populations as their limited foraging range may reduce their access to floral resources in times of increased competition [18]. In Guangxi, *A. florea* and *A. dorsata* have apparently coexisted with *A. cerana* for decades or even centuries, suggesting that coexistence can be sustained. However, the rapid growth of commercial honey bee colonies has only emerged in the past 20 years. It remains unclear whether this large and rapid increase of managed colonies will affect the survival of wild honey bees (*A. florea* and *A. dorsata*). Therefore, in this study, we studied the biological characteristics of *A. florea*, *A. dorsata*, *A. m. ligustica*, and *A. cerana* to analyze the possible competition among them. We also compared the morphometric characteristics of *A. florea* and *A. dorsata* between samples collected in 2012 and 2023 to determine whether their morphometric characteristics were affected by this competition.

Based on the survey results, for all four species, their swarming periods, mating periods, and the emergence of drones highly overlap in spring, with *A. florea* and *A. dorsata* initiating hive activities slightly earlier. Spring is the breeding season for *A. m. ligustica* and *A. cerana* [34], and beekeepers conduct activities such as swarming, queen rearing, and mating during this time. Managed honey bees may occupy an area, forming a drone congregation zone where the altitude, the sunlight, and the airflow are highly favorable for mating. Due to their smaller size, *A. florea* might be at a disadvantage when competing for mating space. It is speculated that the mating success rate of *A. florea* may decline as a result of this competition. However, *A. dorsata* are larger in size, and it remains unclear whether their mating success rate would be affected by the competition for mating space [35]. In autumn, there is another peak in reproduction for *A. cerana* and *A. m. ligustica*, while this does not take place for *A. florea* and *A. dorsata*. This difference may be attributed to beekeepers’ interventions to quickly restore and strengthen the colonies after the hot summer, preparing for the subsequent honey collection. Therefore, during autumn and winter, *A. cerana* and *A. m. ligustica* still maintain strong colony size. This ensures that they maintain a dominant position in foraging during autumn and winter. Based on the survey results, for all four species, the nectar and pollen source plants highly overlap. Inevitably, *A. cerana* and *A. m. ligutica* compete for food resources with *A. florea* and *A. dorsata*, potentially leading to food shortages for the latter two species and hindering the development of wild bee populations. In 2023, the study by MacInnis et al. provided evidence to support that honey bee abundance negatively affects wild bee diversity through exploitative competition for urban floral resources [18]. Our studies also suggest that a high density of commercial bee colonies have an impact on the mating and foraging activities of wild bees.

Through a comparison of the morphologies of *A. florea* and *A. dorsata* between 2012 and 2023, it has been observed that the aspect ratios of the forewings of both *A. florea* and *A. dorsata* have significantly increased. Changes in the aspect ratio would affect the flight capabilities. The larger aspect ratio indicates more flattened wings. It helps insects to maintain stability during high-speed flight, to reduce air resistance, and enhances flight efficiency [36,37,38]. This change in aspect ratio may be attributed to the rapid increase in managed honey bee populations, which has encroached upon the living spaces of *A. florea* and *A. dorsata*. In response to this threat, *A. florea* and *A. dorsata* must rely on faster flight speeds to occupy mating spaces, to implement foraging activities, and to maintain their survival. Additionally, the wax-mirror area of the abdomen of *A. dorsata* has expanded. A larger wax-mirror area enhances their ability to secrete wax and construct combs [39], enabling them to repair their nests more quickly after migration and better adapt to environmental changes.

The comparison of morphological characteristics revealed that the hair length of both *A. florea* and the *A. dorsata* was significantly longer than that of the *A. cerana*, with *A. cerana* having the shortest hair length. The hair on the surface of bees’ body helps it regulating body temperature [40,41]. Honey bees with shorter body hair may exhibit better heat tolerance compared to those with longer hair. Guangxi is a region with relatively high temperatures throughout the year. Although *A. florea*, *A. dorsata*, and *A. cerana* are all native bees in Guangxi, *A. cerana* are managed honey bees. Due to the artificial selection for better heat tolerance, the hair length of *A. cerana* in the region has become shorter. In contrast, *A. florea* and *A. dorsata* are wild honey bees, without selection pressure on hair length. This explains why the hair length of *A. cerana* is shorter than *A. florea*, though *A. cerana* has a larger body size.

Through the analysis of the coefficient of variation, it was found that *A. florea* and *A. dorsata* exhibit higher genetic diversity compared to *A. m. ligustica* and *A. cerana*. *A. florea* and *A. dorsata* are wild honey bees that need to better adapt to environmental changes for the reproduction and development of their populations. Therefore, they retain higher genetic diversity. In contrast, *A. m. ligustica* and *A. cerana* are managed honey bees, and the artificial selection aimed at higher productivity has led to a decline in their genetic diversity.

In the process of agricultural and industrial modernization in China, wild honey bees have suffered a series of adverse effects on their survival. Our results showed that the large and rapid increase in managed honey bees in the Guangxi region has affected the nesting, mating, foraging, and pollination activities of *A. florea* and *A. dorsata.* The morphometric analysis showed that the aspect ratios of the forewings of both *A. florea* and *A. dorsata* have significantly increased in the past ten years, which has helped them to adapt to the competition brought by *A. m. ligustica* and *A. cerana*. The diversity results showed that *A. florea* and *A. dorsata* are not currently facing threats to their survival. However, we need to continuously monitor the diversity of *A. florea* and *A. dorsata*. Preserving wild honey bees is of great significance for ecological balance.

## Data Availability

The original contributions presented in this study are included in the article/supplementary material. Further inquiries can be directed to the corresponding author.

## Supplementary Materials

The following supporting information can be downloaded at: https://www.mdpi.com/article/10.3390/life15030341/s1, Table S1: The mean and standard deviation of morphological indicators for *A. florea*, *A. dorsata*, *A. m. ligustica* and *A. cerana* collected in 2023; Table S2: The mean and standard deviations of morphological indicators for *A. florea* and *A. dorsata* collected in 2012.
