# Supplementary material for: Investigation and Study on the Biology and Morphology of Apis florea and Apis dorsata in Southern China
Source: Life (Basel). 2025 Feb 21;15(3):341. doi: 10.3390/life15030341 (PMC11943900; doi:10.3390/life15030341)
Supplement: Supplementary file 1 [file life-15-00341-s001.zip › Table S1 and S2 - clear.pdf]

Table S1. The mean and standard deviation of morphological indicators for *A. florea*, *A. dorsata*, *A. m. ligustica* and *A. cerana* collected in 2023.

Morphometric characters	*A. florea*	*A. dorsata*	*A. m. ligustica*	*A. cerana*
Pigmentation of tergite 2	7.79±0.49	7.02±1.16	7.41±1.75	7.89±0.1
Pigmentation of tergite 3	6.94±0.57^{ab}	5.87±1.3^{b}	6.98±0.91^{ab}	7.33±0.36^{a}
Pigmentation of tergite 4	3.36±1.08^{c}	4.81±1^{b}	5.55±0.98^{b}	7.34±0.42^{a}
Pigmentation of scutellum, Cupolla	0.04±0.13^{d}	0.89±0.67^{c}	5.19±1.03^{b}	7.89±0.48^{a}
Pigmentation of scutellum, B and K	0.39±0.72^{b}	0.64±0.59^{b}	1.44±0.66^{a}	1.06±0.64^{ab}
Pigmentation of labrum 1	0^{c}	0^{c}	2.64±1.31^{b}	6^{a}
Pigmentation of labrum 2	0^{b}	0^{b}	2.04±0.91^{a}	0^{b}
Width of tomentum on tergite 4	0.39±0.05^{d}	1.78±0.33^{a}	0.93±0.06^{b}	0.74±0.12^{c}
Width of stripe posterior of tomentum	0.6±0.03^{b}	0.12±0.1^{d}	0.49±0.05^{c}	0.78±0.07^{a}
Length of cover hair on tergite 5	0.19±0.02^{ab}	0.19±0.04^{ab}	0.23±0.02^{a}	0.14±0.01^{b}
Proboscis	4.35±0.05^{d}	8.00±0.16^{a}	5.86±0.24^{b}	4.99±0.12^{c}
Femur	1.77±0.02^{d}	3.28±0.09^{a}	2.59±0.05^{b}	2.35±0.05^{c}
Tibia	2.18±0.04^{d}	4.16±0.13^{a}	3.09±0.07^{b}	2.89±0.09^{c}
Basitarsus length	1.36±0.01^{d}	2.94±0.07^{a}	2.01±0.07^{b}	1.81±0.05^{c}
Basitarsus width	0.67±0.02^{d}	1.35±0.06^{a}	1.15±0.03^{b}	1.04±0.03^{c}
Tergite 3, longitudinal	1.52±0.03^{d}	2.91±0.08^{a}	2.13±0.25^{b}	1.79±0.04^{c}
Tergite 4, longitudinal	1.36±0.03^{d}	2.94±0.08^{a}	2.09±0.24^{b}	1.74±0.05^{c}
Sternite 3, longitudinal	1.86±0.02^{d}	4.15±0.12^{a}	2.54±0.06^{b}	2.39±0.04^{c}
Sternite 6, longitudinal	1.48±0.07^{d}	3.31±0.1^{a}	2.55±0.06^{b}	2.34±0.04^{c}
Sternite 6, transversal	1.94±0.04^{d}	3.29±0.12^{a}	2.97±0.13^{b}	2.76±0.05^{c}

Wax mirror of sternite 3 longitudinal	0.86±0.01^{d}	1.84±0.06^{a}	1.4±0.03^{b}	1.14±0.03^{c}
Wax mirror of sternite 3 transversal	1.51±0.03^{d}	2.61±0.1^{a}	2.41±0.06^{b}	2.13±0.03^{c}
Distance between wax mirrors st. 3	0.07±0.01^{d}	0.18±0.03^{c}	0.22±0.01^{b}	0.29±0.02^{a}
Fore wing length	6.91±0.06^{d}	13.71±0.52^{a}	9.12±0.22^{b}	8.36±0.15^{c}
Fore wing width	2.31±0.03^{d}	4.44±0.18^{a}	3.06±0.06^{b}	2.87±0.05^{c}
Cubital vein, distance a	0.54±0.02^{b}	1.17±0.15^{a}	0.56±0.02^{b}	0.52±0.02^{b}
Cubital vein, distance b	0.19±0.01^{b}	0.16±0.01^{c}	0.22±0.02^{a}	0.14±0.01^{d}
Cubital index	2.95±0.25^{c}	7.65±0.65^{a}	2.62±0.31^{c}	3.8±0.23^{b}
Number of hooks	11.57±0.65^{d}	24.53±1.16^{a}	21.98±1.29^{b}	18.55±0.66^{c}
Wing angle A4	33.35±0.92^{b}	39.27±3.13^{a}	28.81±1.21^{d}	31.23±0.85^{c}
Wing angle B4	88.84±2.73^{b}	77.54±2.56^{c}	111.55±3.05^{a}	109±2.65^{a}
Wing angle D7	86.22±1.61^{d}	91.96±1.13^{c}	97.94±1.17^{a}	94.73±1.08^{b}
Wing angle E9	17.95±0.58^{c}	18.2±0.52^{c}	22.61±0.53^{a}	19.51±0.47^{b}
Wing angle G18	101.86±1.56^{a}	101.47±1.63^{a}	92.67±2.49^{c}	88.09±2.31^{d}
Wing angle J10	39±0.87^{c}	35.51±1.2^{d}	52.72±1.17^{a}	48.84±1.79^{b}
Wing angle J16	113.23±1.45^{a}	90±2.89^{c}	88.96±2.04^{c}	102.85±1.94^{b}
Wing angle K19	70.37±1.22^{d}	72.34±1.34^{c}	77.49±1.58^{b}	78.84±1.14^{a}
Wing angle L13	16.2±0.51^{a}	11.64±0.65^{c}	14.08±1.04^{b}	14.21±0.6^{b}
Wing angle N23	73.52±1.49^{c}	73.56±3.68^{c}	86.79±2.25^{a}	82.47±2.51^{b}
Wing angle O26	29.14±1.44^{d}	35.9±1.9^{b}	39.08±1.6^{a}	33.46±1.34^{c}

Note: Values with different superscripts show significant levels within columns: $^{a, b, c, d}$ $P<0.05$.

Table S2. The mean and standard deviations of morphological indicators for *A. florea* and *A. dorsata* collected in 2012.

Morphometric characters	*A. florea*	*A. dorsata*
Femur	--	3.30±0.08
Tibia	--	4.13±0.12
Basitarsus length	--	2.92±0.07
Basitarsus width	--	1.36±0.04
Tergite 3, longitudinal	--	2.90±0.06

Sternite 3, longitudinal	--	3.99±0.07
Sternite 6, longitudinal	--	3.18±0.07
Sternite 6, transversal	--	3.14±0.09
Wax mirror of sternite 3 longitudinal	--	1.64±0.08[a]
Wax mirror of sternite 3 transversal	--	2.49±0.06
Distance between wax mirrors st. 3	--	0.17±0.04
Fore wing length	6.82±0.14	13.36±0.15
Fore wing width	2.36±0.04	4.55±0.08
Cubital vein, distance a	0.56±0.03	1.16±0.06
Cubital vein, distance b	0.18±0.01	0.17±0.02
Wing angle A4	33.41±2.53	39.12±1.91
Wing angle B4	86.65±5.10	76.40±4.07
Wing angle D7	86.77±2.64	89.37±3.37
Wing angle E9	17.47±1.69	17.99±1.21
Wing angle G18	103.16±5.39	104.11±6.19
Wing angle J10	41.29±2.59	35.58±2.86
Wing angle J16	116.11±3.16	87.70±5.58
Wing angle K19	72.88±3.54	72.88±2.35
Wing angle L13	17.54±2.18	13.59±1.76
Wing angle N23	79.78±3.33	68.06±4.90
Wing angle O26	29.33±2.98	39.50±4.93

Note: --, no data.
